# Supplementary material for: Polyphenol Profile and Biological Activity Comparisons of Different Parts of Astragalus macrocephalus subsp. finitimus from Turkey
Source: Biology (Basel). 2020 Aug 17;9(8):231. doi: 10.3390/biology9080231 (PMC7464095; doi:10.3390/biology9080231)
Supplement: Supplementary file 1 [file biology-09-00231-s001.pdf]

# Supplementary Materials

In **section S.1** was given analytical methods applied for phenolic composition, antioxidant and enzyme inhibitory activities.

**Section S.1: Analytical methods applied for phenolic composition, antioxidant and enzyme inhibitory activities.**

**Phytochemical analysis**

An Agilent Technologies 1260 Infinity liquid chromatography system hyphenated to a 6420 Triple Quad mass spectrometer was used for quantitative analyses. Chromatographic separation was carried out on a Poroshell 120 EC-C18 (100 mm × 4.6 mm I.D., 2.7 μm) column. Three mobile phases were tested to obtain a complete resolution of all isomers and the highest sensitivity for all target compounds, namely: (i) 0.1% formic acid/methanol, (ii) 5 mM ammonium acetate/acetonitrile with 0.1% acetic acid and (iii) 10 mM ammonium formate with 0.1% formic acid/acetonitrile with 0.1% formic acid, respectively. The first mobile phase configuration (0.1% formic acid/methanol) was selected on the base of the better chromatographic resolution of isomeric compounds. On the other hand, the selected mobile phase configuration also provided higher sensitivity for many of the phenolic compounds. As a result, the mobile phase was made up from solvent A (0.1%, *v*/*v* formic acid solution) and solvent B (methanol). The gradient profile was set as follows: 0.00 min 2% B eluent, 3.00 min 2% B eluent, 6.00 min 25% B eluent, 10.00 min 50% B eluent, 14.00 min 95% B eluent, 17.00 min 95% B and 17.50 min 2% B eluent. The column temperature was maintained at 25 °C. The flow rate was 0.4 mL $min^{-1}$ and the injection volume was 2.0 μL.

The tandem mass spectrometer was interfaced to the LC system via an ESI source. The electrospray source of the MS was operated in negative and positive multiple reaction monitoring (MRM) mode and the interface conditions were as follows: capillary voltage of −3.5 kV, gas temperature of 300 °C and gas flow of 11 L $min^{-1}$. The nebulizer pressure was 40 psi.

**Table S1.** ESI–MS/MS Parameters and analytical characteristics for the Analysis of Target Analytes by MRM Negative and Positive Ionization Mode.

| Target compounds | Rt (min) | Precursor ion | MRM1 (CE, V) | MRM2 (CE, V) | Linear equation | $R^2$ | LOD (μg/L) | LOQ (μg/L) |
|---|---|---|---|---|---|---|---|---|
| Compounds analyzed by NI mode | | | | | | | | |
| Gallic acid | 8.891 | 168.9 [M − H] − | 125.0 (10) | – | y = 4.82x − 26.48 | 0.9988 | 1.46 | 4.88 |
| Protocatechuic acid | 10.818 | 152.9 [M − H] − | 108.9 (12) | – | y = 5.65x − 9.99 | 0.9990 | 1.17 | 3.88 |
| 3,4-Dihydroxyphenylacetic acid | 11.224 | 167.0 [M − H] − | 123.0 (2) | – | y = 5.13x − 12.39 | 0.9990 | 1.35 | 4.51 |
| (+)-Catechin | 11.369 | 289.0 [M − H] − | 245.0 (6) | 202.9 (12) | y = 1.45x + 1.95 | 0.9974 | 3.96 | 13.20 |
| Pyrocatechol | 11.506 | 109.0 [M − H] − | 90.6 (18) | 52.9 (16) | y = 0.11x − 0.52 | 0.9916 | 9.62 | 32.08 |
| 2,5-Dihydroxybenzoic acid | 12.412 | 152.9 [M − H] − | 109.0 (10) | – | y = 3.79x − 14.12 | 0.9980 | 2.12 | 7.08 |
| 4-Hydroxybenzoic acid | 12.439 | 136.9 [M − H] − | 93.1 (14) | – | y = 7.62x + 22.79 | 0.9996 | 1.72 | 5.72 |
| Caffeic acid | 12.841 | 179.0 [M − H] − | 135.0 (12) | – | y = 11.09x + 16.73 | 0.9997 | 3.15 | 10.50 |
| Vanillic acid | 12.843 | 166.9 [M − H] − | 151.8 (10) | 122.6 (6) | y = 0.49x − 1.61 | 0.9968 | 2.56 | 8.54 |
| Syringic acid | 12.963 | 196.9 [M − H] − | 181.9 (8) | 152.8 (6) | y = 0.74x − 1.54 | 0.9975 | 3.75 | 12.50 |
| 3-Hydroxybenzoic acid | 13.259 | 137.0 [M − H] − | 93.0 (6) | – | y = 3.69x − 12.29 | 0.9991 | 1.86 | 6.20 |
| Vanillin | 13.397 | 151.0 [M − H] − | 136.0 (10) | – | y = 2.02x + 135.49 | 0.9926 | 15.23 | 50.77 |
| Verbascoside | 13.589 | 623.0 [M − H] − | 461.0 (26) | 160.8 (36) | y = 8.59x − 28.05 | 0.9988 | 0.82 | 2.75 |
| Taxifolin | 13.909 | 303.0 [M − H] − | 285.1 (2) | 125.0 (14) | y = 12.32x + 9.98 | 0.9993 | 1.82 | 6.05 |
| Sinapic acid | 13.992 | 222.9 [M − H] − | 207.9 (6) | 163.8 (6) | y = 2.09x − 6.79 | 0.9974 | 2.64 | 8.78 |
| p-Coumaric acid | 14.022 | 162.9 [M − H] − | 119.0 (12) | – | y = 17.51x + 53.73 | 0.9997 | 1.93 | 6.44 |
| Ferulic acid | 14.120 | 193.0 [M − H] − | 177.8 (8) | 134.0 (12) | y = 3.32x − 4.30 | 0.9992 | 1.43 | 4.76 |
| Luteolin 7-glucoside | 14.266 | 447.1 [M − H] − | 285.0 (24) | – | y = 45.25x + 156.48 | 0.9996 | 0.45 | 1.51 |
| Rosmarinic acid | 14.600 | 359.0 [M − H] − | 196.9 (10) | 160.9 (10) | y = 9.82x − 17.98 | 0.9989 | 0.57 | 1.89 |
| 2-Hydroxycinnamic acid | 15.031 | 162.9 [M − H] − | 119.1 (10) | – | y = 16.72x − 26.94 | 0.9996 | 0.61 | 2.03 |
| Pinoresinol | 15.118 | 357.0 [M − H] − | 151.0 (12) | 135.7 (34) | y = 0.80x − 2.69 | 0.9966 | 3.94 | 13.12 |
| Eriodictyol | 15.247 | 287.0 [M − H] − | 151.0 (4) | 134.9 (22) | y = 14.24x − 0.50 | 0.9998 | 0.80 | 2.68 |
| Quercetin | 15.668 | 301.0 [M − H] − | 178.6 (10) | 151.0 (16) | y = 14.68x − 18.25 | 0.9997 | 1.23 | 4.10 |
| Kaempferol | 16.236 | 285.0 [M − H] − | 242.8 (16) | 229.1 (18) | y = 0.82x − 3.06 | 0.9959 | 3.30 | 10.99 |
| Compounds analyzed by PI mode | | | | | | | | |
| Chlorogenic acid | 11.802 | 355.0 [M + H] + | 163.0 (10) | – | y = 12.14x + 32.34 | 0.9995 | 0.55 | 1.82 |
| (−)-Epicatechin | 12.458 | 291.0 [M + H] + | 139.1 (12) | 122.9 (36) | y = 9.11x − 9.99 | 0.9971 | 1.85 | 6.18 |
| Hesperidin | 14.412 | 611.1 [M + H] + | 449.2 (4) | 303.0 (20) | y = 5.98x + 0.42 | 0.9993 | 1.73 | 5.77 |
| Hyperoside | 14.506 | 465.1 [M + H] + | 303.1 (8) | – | y = 16.32x − 1.26 | 0.9998 | 0.99 | 3.31 |
| Apigenin 7-glucoside | 14.781 | 433.1 [M + H] + | 271.0 (18) | – | y = 21.33x − 31.69 | 0.9983 | 0.41 | 1.35 |
| Luteolin | 15.923 | 287.0 [M + H] + | 153.1 (34) | 135.1 (36) | y = 8.96x + 26.80 | 0.9992 | 1.34 | 4.46 |
| Apigenin | 16.382 | 271.0 [M + H] + | 153.0 (34) | 119.1 (36) | y = 11.29x + 38.05 | 0.9987 | 0.96 | 3.20 |

$R_t$, retention time; NI, negative ion; and PI, positive ion. LOD and LOQ: limit of detection and limit of quantification, respectively.

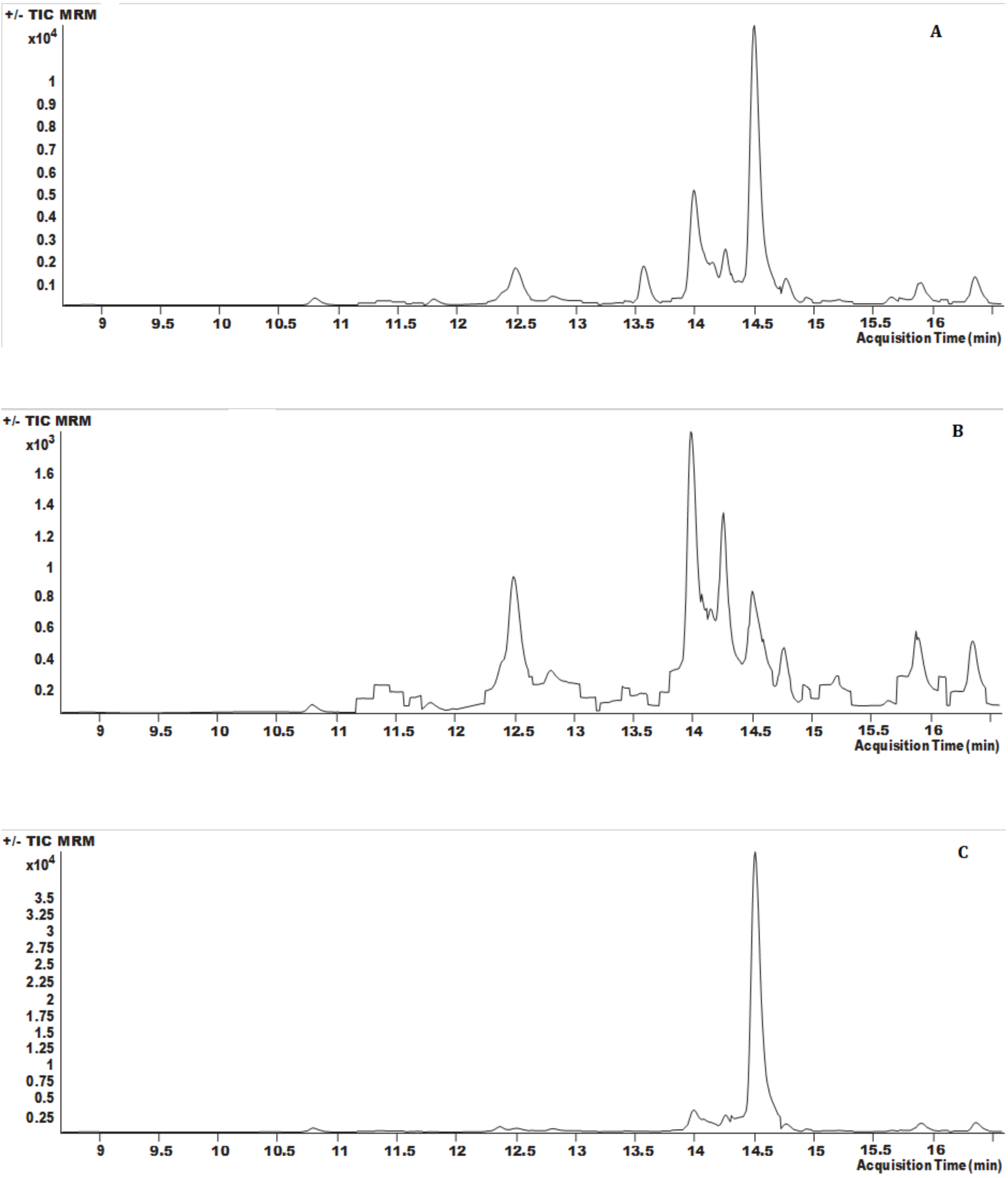
+/- TIC MRM
x10^4
A
1
0.9
0.8
0.7
0.6
0.5
0.4
0.3
0.2
0.1
9
9.5
10
10.5
11
11.5
12
12.5
13
13.5
14
14.5
15
15.5
16
Acquisition Time (min)
+/- TIC MRM
x10^3
B
1.6
1.4
1.2
1
0.8
0.6
0.4
0.2
9
9.5
10
10.5
11
11.5
12
12.5
13
13.5
14
14.5
15
15.5
16
Acquisition Time (min)
+/- TIC MRM
x10^4
C
3.5
3.25
3
2.75
2.5
2.25
2
1.75
1.5
1.25
1
0.75
0.5
0.25
9
9.5
10
10.5
11
11.5
12
12.5
13
13.5
14
14.5
15
15.5
16
Acquisition Time (min)

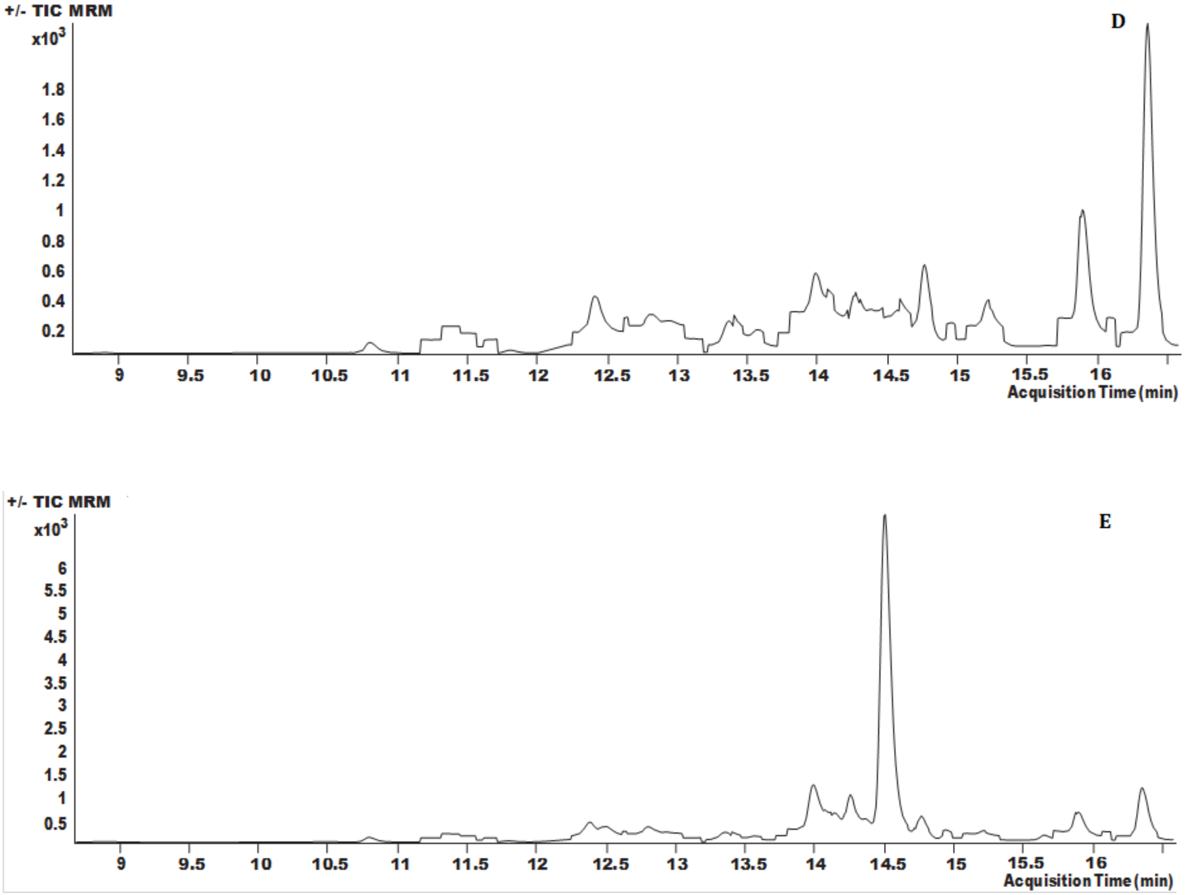


**Figure S1.** LC-ESI-MS/MS chromatograms of the methanol extracts from aerial parts (**A**), flowers (**B**), leaves (**C**), roots (**D**), and stems (**E**) of *A. macrocephalus* subsp. *finitimus*.

### Assays for total phenolic and flavonoid content

For total phenolic content, sample solution (0.25 mL) was mixed with diluted Folin-Ciocalteu reagent (1 mL, 1:9) and shaken vigorously. After 3 min, $Na_2CO_3$ solution (0.75 mL, 1%) was added and the sample absorbance was read at 760 nm after 2 h incubation at room temperature. Total phenolic content was expressed as equivalents of gallic acid.

For total flavonoid content, sample solution (1 mL) was mixed with the same volume of aluminium trichloride (2%) in methanol. Similarly, a blank was prepared by adding sample solution (1 mL) to methanol (1 mL) without $AlCl_3$. The sample and blank absorbance were read at 415 nm after 10 min incubation at room temperature. Absorbance of the blank was subtracted from that of the sample. Total flavonoid content was expressed as equivalents of quercetin.

### Biological activity

Total antioxidant activity of the samples was evaluated by phosphomolybdenum method. Sample solution (0.2 mL) was combined with 2 mL of reagent solution (0.6 M sulfuric acid, 28 mM sodium phosphate and 4 mM ammonium molybdate). The sample absorbance was read at 695 nm after 90 min incubation at 95 °C.

For 1,1-diphenyl-2-picrylhydrazyl (DPPH) radical scavenging activity, sample solution (1 mL) was added to a 4 mL of 0.004% methanol solution of DPPH. Sample absorbance was read at 517 nm after 30 min incubation at room temperature in dark.

For ABTS cation radical scavenging activity, briefly, $ABTS^{\cdot +}$ radical cation was produced directly by reacting 7 mM ABTS solution with 2.45 mM potassium persulfate and allowing the mixture to stand for 1216 h in dark at the room temperature. Prior to beginning the assay, ABTS solution was diluted with methanol to obtain an absorbance of 0.700 ± 0.02 at 734 nm. Sample solution (1 mL) was added to ABTS solution (2 mL) and mixed. Sample absorbance was read at 734 nm after 7 min incubation at room temperature.

For metal chelating activity on ferrous ions, briefly, sample solution (2 mL) was added to $FeCl_2$ solution (0.05 mL, 2 mM). The reaction was initiated by the addition of 5 mM ferrozine (0.2 mL). Similarly, a blank was prepared by adding sample solution (2 mL) to $FeCl_2$ solution (0.05 mL, 2 mM) and water (0.2 mL) without ferrozine. Then, the sample and blank absorbance were read at 562 nm after 10 min incubation at room temperature.

For cupric ion reducing activity (CUPRAC), sample solution (0.5 mL) was added to a premixed reaction mixture containing $CuCl_2$ (1 mL, 10 mM), neocuproine (1 mL, 7.5 mM) and $NH_4Ac$ buffer (1 mL, 1 M, pH 7.0). Similarly, a blank was prepared by adding sample solution (0.5 mL) to a premixed reaction mixture (3 mL) without $CuCl_2$. Then, the sample and blank absorbance were read at 450 nm after 30 min incubation at room temperature.

For ferric reducing antioxidant power (FRAP), sample solution (0.1 mL) was added to a premixed FRAP reagent (2 mL) containing acetate buffer (0.3 M, pH 3.6), 2,4,6-tris(2-pyridyl)-s-triazine (TPTZ) (10 mM) in 40 mM HCl and ferric chloride (20 mM) in a ratio of 10:1:1 (*v*/*v*/*v*). Then, the sample absorbance was read at 593 nm after 30 min incubation at room temperature.

Inhibitory activity on $\alpha$-amylase was performed using Caraway-Somogyi iodine/potassium iodide (IKI) method. Sample solution (25 μL) was mixed with $\alpha$-amylase solution (50 μL) in phosphate buffer (pH 6.9 with 6 mM sodium chloride) in a 96-well micro plate and incubated for 10 min at 37 °C. After pre-incubation, the reaction was initiated by the addition of starch solution (50 μL, 0.05%). Similarly, a blank was prepared by adding sample solution to all reaction reagents without enzyme solution ($\alpha$-amylase). The reaction mixture was incubated 10 min at 37 °C. The reaction was then stopped with the addition of HCl (25 μL, 1 M). This was followed by the addition of iodine-potassium iodide solution (100 μL). The sample and blank absorbance were read at 630 nm. Absorbance of the blank was subtracted from that of the sample.

Tyrosinase inhibitory activity was measured using a modified dopachrome method with L-DOPA as substrate. Sample solution (25 μL) was mixed with tyrosinase solution (40 μL) and phosphate buffer (100 μL, pH 6.8) in a 96-well microplate and incubated for 15 min at 25 °C. The reaction was then initiated with the addition of *L*-DOPA (40 μL). Similarly, a blank was prepared by adding sample solution to all reaction reagents without enzyme (tyrosinase) solution. The sample and blank absorbance were read at 492 nm after 10 min incubation at 25 °C.

The sample concentration, which decreases the initial concentration by 50% for enzyme inhibition, radical scavenging and metal chelation tests, was defined as $IC_{50}$, while the $EC_{50}$ values were calculated as sample concentration providing 0.500 absorbance for reducing power and phosphomolybdenum assays. The biological activities of the extracts were also expressed as mg standard equivalent/g extract and compared with those of the standards, including trolox, ethylenediaminetetraacetic acid (disodium salt) (EDTA), kojic acid, and acarbose, used as positive controls.
